# Treatment of patients with aortic atherosclerotic disease with paclitaxel-associated lipid nanoparticles

**DOI:** 10.6061/clinics/2016(08)05

**Published:** 2016-08

**Authors:** Afonso A. Shiozaki, Tiago Senra, Aleksandra T. Morikawa, Débora F. Deus, Antonio T Paladino, Ibraim M.F. Pinto, Raul C. Maranhão

**Affiliations:** I Instituto Dante Pazzanese de Cardiologia, São Paulo/SP, Brazil; II Laboratório de Metabolismo e Lípides - Instituto do Coração (InCor) do Hospital das Clínicas da Faculdade de Medicina da Universidade de São Paulo, São Paulo/SP, Brazil; III Hospital Maringá, Maringá/Paraná, Brazil; IV Faculdade de Ciências Farmacêuticas da Universidade de São Paulo, São Paulo/SP, Brazil

**Keywords:** Nanoparticles, Solid Lipid Particles, Taxanes, Cardiovascular Disease Treatment, LDL Receptors, Drug Targeting

## Abstract

**OBJECTIVE:**

The toxicity of anti-cancer chemotherapeutic agents can be reduced by associating these compounds, such as the anti-proliferative agent paclitaxel, with a cholesterol-rich nanoemulsion (LDE) that mimics the lipid composition of low-density lipoprotein (LDL). When injected into circulation, the LDE concentrates the carried drugs in neoplastic tissues and atherosclerotic lesions. In rabbits, atherosclerotic lesion size was reduced by 65% following LDE-paclitaxel treatment. The current study aimed to test the effectiveness of LDE-paclitaxel on inpatients with aortic atherosclerosis.

**METHODS:**

This study tested a 175 mg/m^2^ body surface area dose of LDE-paclitaxel (intravenous administration, 3/3 weeks for 6 cycles) in patients with aortic atherosclerosis who were aged between 69 and 86 yrs. A control group of 9 untreated patients with aortic atherosclerosis (72-83 yrs) was also observed.

**RESULTS:**

The LDE-paclitaxel treatment elicited no important clinical or laboratory toxicities. Images were acquired via multiple detector computer tomography angiography (64-slice scanner) before treatment and at 1-2 months after treatment. The images showed that the mean plaque volume in the aortic artery wall was reduced in 4 of the 8 patients, while in 3 patients it remained unchanged and in one patient it increased. In the control group, images were acquired twice with an interval of 6-8 months. None of the patients in this group exhibited a reduction in plaque volume; in contrast, the plaque volume increased in three patients and remained stable in four patients. During the study period, one death unrelated to the treatment occurred in the LDE-paclitaxel group and one death occurred in the control group.

**CONCLUSION:**

Treatment with LDE-paclitaxel was tolerated by patients with cardiovascular disease and showed the potential to reduce atherosclerotic lesion size.

## INTRODUCTION

Severe atherosclerotic aortic disease is a life-threatening condition that markedly increases the risks of thromboembolic accidents such as stroke, acute myocardial infarction and obstruction of peripheral vessels, especially in elderly subjects with large, complex lesions 1,2. In this setting, the use of non-invasive energetic treatments to reduce lesion size and inflammation is essential for the prevention of subsequent cardiovascular events.

The most potent anti-proliferative drugs currently available are chemotherapeutic agents used for cancer treatment. However, the systemic use of these drugs at high doses for the treatment of atherosclerotic cardiovascular diseases 3 is unlikely due to their significant, often life-threatening toxicity. Nonetheless, the toxicity of such agents can be strongly diminished by the use of optimized drug-delivery systems. In a pioneer study performed on patients with acute leukemia, Maranhão et al. 4 reported the potential of a cholesterol-rich non-protein nanoemulsion (LDE) as a drug targeting agent. LDE particles have lipid compositions and structures that resemble low-density lipoprotein (LDL) and can be injected directly into the bloodstream. When LDE particles come into contact with plasma, they acquire exchangeable apolipoproteins from native lipoproteins, such as apolipoprotein (apo) E, which binds the particles to LDL receptors 5. In neoplastic cells, lipoprotein receptors are overexpressed 6, such that uptake of native LDL and of LDE particles is increased relative to that in normal tissues. It was also shown that the uptake of LDE particles by the aortas of cholesterol-fed rabbits is increased in comparison to normal aortas 7 and that rabbit-grafted hearts take up the nanoemulsion at amounts fourfold greater than native hearts 8.

When tested in mice by measuring classical pharmacological parameters, the toxicity of paclitaxel was markedly reduced upon association of the drug with LDE particles. The LD50 of LDE-paclitaxel was tenfold higher than that of a commercial preparation that uses Cremophor EL as a drug delivery vehicle 9. Subsequently, patients with advanced solid cancers treated with LDE-paclitaxel at the high doses used in clinical practice did not show significant clinical or laboratory toxicity 10.

LDE-paclitaxel treatment of rabbits induced to exhibit atherosclerosis via high cholesterol intake resulted in a 65% reduction in lesion size 7. In rabbits that underwent heterotopic heart transplantation, LDE-paclitaxel treatment markedly reduced heart graft damage by preventing coronary vessel destruction and macrophage invasion into the myocardium 8.

The low toxicity of LDE-paclitaxel shown in cancer patients 10 and the convincing anti-atherosclerotic effects of this preparation on the aortic lesions of cholesterol-fed rabbits 7 prompted us to design the current pilot clinical study. The aim of this study was to investigate whether patients with aortic atherosclerotic disease showed good tolerability to high-dose LDE-paclitaxel treatment and whether this formulation could achieve reductions in plaque area that could be documented using a non-invasive, angiotomography-based approach. To the best of our knowledge, this is the first study in which patients with cardiovascular disease were treated with a systemic chemotherapeutic scheme used for cancer treatment.

## METHODS

### Patients

The study group included consecutive patients referred for computerized tomography of the aorta at the Cardiovascular Computed Tomography Section of Dante Pazzanese Cardiology Institute in the city of São Paulo and at the Diagnosis Center of the Paraná Hospital in the city of Maringá. The recruited patients were on clinical follow-up due to aortic disease. The inclusion was a diagnosis of severe aortic atherosclerosis (plaque thickness >4 mm) by multidetector computed tomography (MDCT) angiography. Ten volunteers were enrolled in the study for treatment with LDE-paclitaxel. Seven of the volunteers were male and three were female. They ranged in age from 69 to 86 years and all showed aortic dilations and ulcers on angiography. All had systemic arterial hypertension, 7 had type 2 diabetes mellitus, 3 had dyslipidemia and 3 were ex-smokers. All were using statins.

A control group (without LDE-paclitaxel treatment) of nine consecutive volunteer patients composed of 8 men and one woman aged 72-84 years with severe aortic atherosclerosis diagnosed by MDCT angiography was also studied. All had systemic arterial hypertension and dyslipidemia, 4 had type 2 diabetes mellitus and 5 were ex-smokers. All were using statins.

Patients with renal dysfunction defined as creatinine clearance <60 mL/min, or with aortic dissection were excluded from both groups. The study protocol was approved by the medical ethics committees of the Dante Pazzanese Cardiology Institute and Paraná Hospital and all participants provided informed written consent.

### Preparation of LDE particles containing paclitaxel oleate

LDE particles containing paclitaxel oleate were prepared by high-pressure homogenization from a pre-emulsion obtained by ultrasonic irradiation until complete drug dissolution, according to previously described methods 11. After the homogenization cycles, the formed emulsion was centrifuged, sterilized by passage through a 0.22-μm pore polycarbonate filter (Millipore, Billerica, MA) and kept at 4°C until use. The final concentration of paclitaxel oleate LDE particles was confirmed by high-performance liquid chromatography (Shimadzu, Columbia, ML), which indicated a yield of association of paclitaxel oleate to the LDE particles of >95%. Based on measurements made by a laser scattering technique 12, the average particle diameter was 52 nm.

### Treatment of patients with LDE-paclitaxel particles

Administration of the treatment commenced at approximately 10:00 a.m. The patients were infused via the brachial vein over an approximate 90-min time course with 175 mg/m^2^ LDE-paclitaxel dissolved in a 0.9% saline solution to a total volume of 250 ml. The patients were clinically monitored for adverse reactions during the infusion and for 1 hour after the procedure. The treatment was administered every 3 weeks for a total of 6 cycles. Clinical and laboratory evaluations were performed 2 weeks after each cycle and the patients could contact the researchers anytime to report symptoms. Toxicity ratings based on NCI Common Toxicity Criteria were assessed before each treatment cycle and treatment was delayed or suspended if a patient experienced serious adverse reactions or if significant laboratory changes were detected. The evaluated laboratory parameters included red and white blood cell counts, platelet counts, electrolytes, cholesterol, triglycerides, apolipoproteins A-I and B, C-reactive protein, liver and renal function and coagulation tests.

### Imaging studies

In the group of patients treated with LDE-paclitaxel, MDCT angiography was performed before treatment initiation and again 1-2 months after the last treatment cycle. In the control group of patients, MDCT angiography was performed twice with an interval of 6-8 months.

Images were acquired using a 64-slice MDCT scanner (Aquilion 64; Toshiba Medical Systems, Otawara, Japan), which is a 64x0.5 mm collimation scanner with a gantry rotation speed of 400 ms/rotation. Scanning was performed at 100 kV and 400-500. Depending on each patient’s body mass index (BMI), the table feed was 6.4 mm/gantry rotation with a beam pitch of 0.2. For thoracic aorta scans, a retrospective electrocardiogram (ECG)-gated protocol with dose modulation was adopted; abdominal aorta scans were performed using a non-gated protocol. The patients received 100 ml intravenous iodinated contrast (iopamidol 370 mg/dl) or a maximum dose of 2 ml/kg.

Atherosclerotic plaques were quantified along the aorta by planimetry performed on 1-mm-thick axial images from pre- and post-treatment scans, such that an exact comparison of the plaque measurements before and after treatment could be performed. Plaque volume was independently quantified by two trained imaging technicians who were blinded to whether the images were taken pre- or post-treatment. Variations in the measurements of plaque volume between pre- and post-treatment were considered accurate when <3%.

### Statistical analysis

Bland-Altman analysis was performed using Prism 6 GraphPad software (San Diego, CA) to verify that agreement existed in the plaque volume measurements taken by the two observers. The differences between the pre- and post-treatment values of plaque volume were compared using a paired Student’s t test. Results were considered significant at the <0.05 level.

## RESULTS

A total of 59 treatment cycles were performed and analyzed with respect to toxicity parameters. Eight patients completed the 6 scheduled treatment cycles without showing appreciable clinical or laboratory toxicity. A 78-year-old male patient experienced sudden death after the fifth treatment cycle. The patient had high cardiovascular risk and was found in his home under cardiopulmonary arrest and transported to the nearest hospital where his death was registered. Necropsy was not performed due to family refusal. This patient had not shown any clinical or laboratory toxicities at any point during the treatment period, and his death was probably unrelated to the intervention. Another patient did not return for the post-treatment angiography within the pre-established period of 1-2 months after the last (6^th^) treatment cycle.

None of the patients showed any common toxicities associated with taxane use, i.e., myelosuppression, nausea, vomiting, alopecia, arthralgia, myalgia or peripheral neuropathy, or any other toxicities (Table 1). Mild leukopenia (white blood cell <4300/µl) was observed after the second and sixth cycles in one patient who remained asymptomatic. Isolated elevation of alkaline phosphatase (AP) and gamma-glutamyl transpeptidase (GGT) was found in one patient throughout the treatment. This patient’s baseline levels of AP and GGT were already elevated at study entry and increased by 1.5- and 4-fold, respectively. Bilirubin, AST and ALT levels; coagulation test results; and liver ultrasound remained normal in all patients throughout the treatment period. Mild alterations in renal function that did not require intervention were observed in up to 3 patients after treatment cycles 1, 2, 3, 5 and 6 (Table 1).

Total, LDL and HDL cholesterol, triglycerides and apolipoprotein A1 and B concentrations did not significantly change during the treatment cycles (Table 2).

Table 3 displays the individual data on aortic wall thickness volume of the eight patients in whom pre- and post-treatment angiographic exams were performed, as measured by the two blinded observers. Bland-Altman analysis showed that there was a strong correlation between the plaque volume measurements of the two observers, with a <3% variation. After treatment with LDE-paclitaxel, plaque volume was reduced by >3% in four patients (patients 1, 4, 5 and 7), remained stable in two patients (patients 3 and 8) and increased by >3% in two patients (patients 2 and 6). However, the differences between the pre- and post-treatment values were not significant (*p*=0.348).

In Table 4, the plaque volume data obtained from the non-treated control patients at 6-8 month intervals are shown. In this group of nine patients, no one showed a decrease in plaque volume over the period between the first and the last observation. In 6 patients, the plaque volumes were stable and in 3 there was a >3% increase in plaque volume. Comparing the data from the non-treated group, there was a statistically significant increase in plaque volume from the first to the second exam (*p*<0.05).

## DISCUSSION

Aortic atheroma is a manifestation of systemic atherosclerosis in which the presence of aortic plaques is associated with the presence of carotid artery or coronary artery plaques. Thus, the identification of aortic atheromas is a strong predictive risk factor for major cardiovascular events, such as stroke or acute myocardial infarction 13,14. In this context, the risk factors for aortic atheroma are similar to those for coronary or cerebrovascular artery atherosclerotic disease, namely, older age and male gender, smoking, hypercholesterolemia, hypertension and diabetes 15. Patients with aortic atherosclerotic disease also seem to benefit from statin use 16.

A 175 mg/m^2^ body surface area paclitaxel dose was tested in the patients with aortic atheroma, which is equal to that used in cancer chemotherapy schemes. Paclitaxel is a major drug in cancer chemotherapy and is also used in pharmacological stents to inhibit restenosis. The mechanism of action for the drug is based on the ability of taxanes to increase tubulin polymerization into stable microtubules. It also promotes microtubule stabilization, thus preventing depolymerization. The overall effect is inhibition of the mitotic apparatus formation and arrest of mitosis in the G2/M phase of the cell cycle 17,18.

The toxicity of commercially available paclitaxel, in which Cremophor EL is used as a drug delivery vehicle, is cumulative. The main toxicities are hematological, with neutropenia being the most prominent toxicity of the drug. Neurotoxicity, arrhythmias and alopecia are other frequent toxicities elicited by paclitaxel, together with major hypersensitivity reactions related to Cremophor EL.

The fact that the toxicity of commercial paclitaxel is cumulative and that paclitaxel-associated LDE particles were administered here at a dose roughly twice that used in the study reported by Margolis et al. 19 for six cycles without producing appreciable toxicities highlights the superior tolerability of LDE-paclitaxel. To document this, a total 59 chemotherapy cycles were analyzed and the results confirmed the absence of the significant toxicities previously shown in cancer patients. This reduced toxicity can be ascribed to several factors, such as the unique biodistribution pattern created by the carrier, the envelopment of the drug in circulation, which diminishes contact with normal tissues and organs and the increased half-life of the drug.

In a previous study, treatment of rabbits harboring atherosclerotic lesions induced by high cholesterol consumption with LDE-paclitaxel markedly decreased macrophage and smooth muscle cell invasion of the intima, as well as the presence of apoptotic cells 7. This accounted for the great reduction in lesion area and intima width observed in the aortas of the treated rabbits. In rabbits with heart grafts, the expression levels of pro-inflammatory factors such as tumor necrosis factor-a, interleukin-1b, interleukin-18, monocyte chemotactic protein-1, vascular cell adhesion molecule-1, matrix metalloproteinase-9, and matrix metalloproteinase-12, as well as the anti-inflammatory factor interleukin-10, were strongly up-regulated in the heart grafts of non-treated animals compared with native hearts 8. Treatment with LDE-paclitaxel resulted in a several fold reduction of the gene expression of both pro- and anti-inflammatory factors in the grafted hearts 20. It is worthwhile to note that similar results were achieved following the treatment of cholesterol-fed rabbits with other preparations of chemotherapeutic agents associated with LDE, such as LDE-etoposide 21 and LDE-methotrexate 22. Thus, it can be assumed that lesion size reduction, as was observed in patients 1, 4, 5 and 7 in the current study, is a consequence not only of the anti-proliferative actions of the drug but also of the reduction of inflammation in the lesion area. It is also worth noting that the inflammatory process increases the actions of proteinases, which can result in plaque rupture and hemorrhage, thrombus formation and thromboembolic accidents.

The large caliber and thickness of the aortic vessel facilitates the quantification of lesion volumes using MDCT, which is non-invasive. The reliability of the quantitative analyses that were independently made by the two blinded imaging technicians was confirmed by the <3% inter-observer variability. Although the study was limited by the sample containing only eight patients, it is noteworthy that four of the patients showed a regression in aortic plaque volume, whereas none of the patients in the non-treated control group showed a regression in lesion size. Additionally, although there was no significant reduction in plaque volume in the LDE-paclitaxel group after treatment, in the control group, probably due to the small number of patients, there was an increase in plaque volume, suggesting disease progression in these patients.

With respect to the data on plasma lipid and apolipoprotein concentrations collected during the study, the lack of observed effect following the LDE-paclitaxel treatment was expected because paclitaxel does not interfere with plasma lipid metabolism. Indeed, the natural history of aortic atheroma is not always progressive; rather, a slow progression in plaque thickness is the most common clinical course 1,2.

This pilot study showed that treatment with high-dose LDE-paclitaxel had low enough toxicity to permit its use in patients with cardiovascular disease. Although not statistically significant, there was an average 18% reduction in plaque volume in four out of the eight participants, which is a promising finding. This result was especially noteworthy in view of the short 18-week treatment period and when considering that plaque reduction did not occur in any of the control group patients. In contrast, statistically significant disease progression was observed in the non-treated control patients. The results of the current study are supportive for future trials aiming to confirm the efficacy of this novel treatment in a large number of participants.

## AUTHOR CONTRIBUTIONS

Shiozaki AA researched the study design, wrote the manuscript and executed the work. Senra T and Paladino-Filho AT executed the work, interpreted the data and performed case selection. Morikawa AT and Dus DF. Pinto IM performed data interpretation and case selection. Maranhão RC researched the study design and helped writing the manuscript.

## Figures and Tables

**Table 1:** Toxicity of LDE-paclitaxel administered as an intravenous infusion over 2 h every 3 weeks to 10 patients with aortic atheroma at a dose of 175 mg/m^2^. Toxicity was classified from grade 1 (mild; asymptomatic or mild symptoms; clinical or diagnostic observations only; intervention not indicated) to grade 5 (death related to adverse event). The data represent the number of patients who presented grade 1 toxicity only because no grade 2-5 toxicities were observed.

Treatment cycles	1	2	3	4	5	6
Grade 1 toxicity						
Clinical						
Nausea	0	0	0	0	0	0
Vomiting	0	0	0	0	0	0
Local pain	0	0	0	0	0	0
Arrhythmia	0	0	0	0	0	0
Fever	0	0	0	0	0	0
Dyspnea	0	0	0	0	0	0
Alopecia	0	0	0	0	0	0
Hematologic						
Anemia	0	0	0	0	0	0
Leucopenia	0	1	0	0	0	1
Thrombocytopenia	0	0	0	0	0	0
Hepatic						
AST	0	0	0	0	0	0
ALT	0	0	0	0	0	0
Bilirubin	0	0	0	0	0	0
AP	1	1	1	1	1	1
GGT	1	1	1	1	1	1
Renal						
Urea	2	2	1	0	1	0
Creatinine	0	3	2	0	0	1

AST: aspartate aminotransferase; ALT: alanine aminotransferase; AP: alkaline phosphatase; GGT: gamma-glutamyl transferase

**Table 2:** Fasting serum lipids and apolipoproteins (in mg/dl) in the patients treated with LDE-paclitaxel, measured approximately 2 weeks after each treatment cycle (the results are shown as the mean±S.D.). Paclitaxel-LDE was administered via intravenous infusion every 3 weeks at a dose of 175 mg/m^2^ body surface area.

Parameters	Treatment Cycles						
	0	1	2	3	4	5	6
Cholesterol (mg/dL)							
Total	167±44	169±32	169±30	168±24	165±28	173±34	204±48
LDL	90±34	90±15	86±14	92±18	87±18	92±22	109±34
HDL	45±14	46±13	49±13	48±14	46±11	47±13	49±12
Triglycerides (mg/dL)	170±93	161±105	124±46	147±56	134±58	156±72	204±76
Apolipoproteins (mg/dL)							
A1	136±19	127±24	131±21	131±9	136±25	136±20	155±20
B	92±30	86±15	86±19	87±20	84±18	89±23	99±38

**Table 3:** Aortic plaque volumes in 8 patients with aortic atherosclerotic disease before and after six cycles of treatment with LDE-paclitaxel administered via intravenous infusion over 2h every three weeks at a 175 mg/m^2^ body surface area dose.

Patient	Plaque Volume (cm^3^)		% Variation
	Pre-treatment	Post-treatment	
1	367	288	-22
2	819	851	+4
3	627	641	+2
4	793	572	-28
5	530	477	-10
6	457	530	+16
7	398	377	-5
8	257	256	0

**Table 4:** Plaque volume (cm^3^) measured twice in the aorta with a 6-8 month interval between the two observations in 9 patients with aortic atherosclerotic disease who were not treated with LDE-paclitaxel (controls).

Patient	Plaque Volume (cm^3^)		% Variation
	1^st^ Observation	2^nd^ Observation	
9	202	204	+2
10	358	363	+2
11	133	136	+2
12	272	280	+3
13	382	398	+4
14	538	552	+3
15	138	142	+3
16	190	201	+6
17	392	406	+4
